# Book Reviews

**Published:** 1809-10

**Authors:** 


### CRITICAL ANALYSIS
OF THE
### RECENT PUBLICATIONS
ON THE
### DIFFERENT BRANCHES OF PHYSIC, SURGERY, AND MEDICAL PHILOSOPHY.

---

### Edinburgh Journal, No. XIX.

( Concluded from our last, pp. 152—166. )

We copy the following, to give Dr. Wilson the credit of a very fair remark on the flimsy basis of some of Bichât's opinions, which are not only unfounded, but for which he has not even the credit of originality.

" The following digression, as connected with the subject before us, will I hope be excused. Bichât has adopted an ingenious opinion of the late Dr. Johnstone, of this place, that the cause of certain muscles being subjected to the will, and others independent of it, is, that the former are supplied with nerves directly from the brain, the latter with nerves passing through ganglia. It is surprising that Bichât should adopt this opinion, while he admits that the observation on which it is founded does not hold in every instance; for it is evident that, if it fails in any one, we must look out for some other explanation of the phenomena."

" But," continues Dr. Wilson, " it does not seem necessary to have recourse to this, or any other hypothesis, to explain why certain muscles are subjected to the will, and others independant of it, if we find that, in every instance, the former are excited by a stimulus communicated through the brain, the latter by a stimulus directly applied to the muscles themselves; for even a muscle of voluntary motion, if the stimulus which excites it be directly applied to the muscle itself, acts involuntarily. If we sprinkle salt on the denuded muscles of a limb, or wound them with an instrument, they are thrown into involuntary contractions. We shall find, on inquiry, that this is really the case; that all the muscles of voluntary motion are excited through the medium of the brain; of involuntary motion, by stimuli immediately applied to the muscles themselves."

Thus, then, muscles of voluntary motion, act involuntary if a stimulus be immediately applied to them; nor does this arise from any consciousness conveyed to the brain, for this action is excited even when the limb is removed from the trunk. What then, we would ask, has the death of the brain to do with the life or death of these muscles? As long as the brain retains its proper functions, and the muscles live, each retaining its communication with the

 other, other, so long the *actions* of these muscles may be expected to be regular, and no longer.

But can we wonder if the voluntary muscles act irregularly from any violence done to the brain, which, whether the seat of volition or not, we find under every derangement produces an effect on the will? Or can we be surprised if the heart and peristaltic motion are little affected by such derangement of the brain, when we know that the muscles of the heart and intestines act without any immediate concurrence of the will? But what has this to do with the *death* of the brain?

The three remaining opinions, we are told, the reader will find considered at length in the first volume of Dr. Wilson's Treatise on Febrile Diseases, the first edition of which was published the same year that Bichât's work appeared at Paris.

It has often been a matter of surprise, that the same discoveries should occur at the same time to different philosophers, in different parts of the world. But considering the communication of knowledge, and the steps by which the establishment of one fact leads to another, this is less remarkable than that men in different countries, should, at the same time, be led to the adoption of physiological enigmas on the same parts, and nearly in the same language.

For the sake of perspicuity, we shall repeat the 3d and 4th propositions, opinions, or whatever these gentlemen may please to call them.

"3. That the functions of life may be divided into *organic* and animal; that is, in more common language, vital and animal; of which the latter are subject to constant intermissions and renewal, their *organs* requiring intervals of rest; the former incessant, their *organs* being endowed with an excitability, which is never impaired, except by disease or death.

"4. That organic contractility, that is, vital excitability, has no relation to a centre, but exists in every part, independently of its existence in any other; while animal contractility, that is, animal excitability, in every part, depends on the existence of a common centre."

In the beginning of the first of these paragraphs, life is divided into *organic* and *animal*, and at the close of the same, it appears that both are *organic*; consisting, one of them, of a set of *organs*, which require intervals of rest: the other, of *organs*, whose *excitability* is never impaired but by disease or death. Without attending to the inaccuracy of terms, even the distinction of properties is inaccurate, for the heart has its alternate rest as well as its action; its diastole being a state of inaction, and its systole of action, or to use common language, of dilatation and contraction,* requiring intervals of rest. Thus, though the functions of this vital part are incessant, its actions are not.

If

* See Hunter, ut supra.

If the excitability of these vital organs is never impaired but by disease or death, the same may be said of the animal, for all the muscles of an animal may be excited to act by a sufficient stimulus, long after they are separated from the brain; in short, as long as they retain life: and if this excitability becomes gradually more feeble, the same may be said of the heart.

The 4th proposition is one of the most ingenious little puzzles of all the rest.

"Organic contractility, that is, vital excitability, has no relation to a centre."

It is certain that those organs which are immediately and constantly necessary for the purposes of life, act without any reference to the brain, or common sensorium, whatever it may be. But have they not a general relation to the parts immediately connected with them, and from thence to the whole? The heart is so constituted, that whilst the lungs perform their functions, and the order of circulation is uninterrupted, its actions are regular. But any cessation, or irregularity in these, produces an irregularity or cessation in the actions of the heart. Thus the heart, which is more particularly instanced as a vital organ, has a relation to a common centre, or to a circle, and for its customary actions requires its customary stimulus. Independently of this, it may be excited into action by a stimulus immediately applied to itself, in the same manner as any voluntary muscle; "whilst animal contractility, continues the proposition, that is, animal excitability, depends upon a common centre." It is true, the muscles of voluntary action require a communication with the brain, in order to act in conformity with the will, by which we suppose is meant the "common centre;" but these, as we before observed, may be excited into action without any kind of connection with the brain, or any other part.

A muscle taken from a living animal may be made to contract; "even a muscle," says Dr. W. "of voluntary motion, if the stimulus which excites it be directly applied to the muscle itself, acts involuntary." This sentence is intelligible; but immediately follows, "all the muscles of voluntary motion are excited through the medium of the brain, of involuntary motion by stimuli immediately applied to the muscles themselves." Yet we have just seen, that either of them may be excited by stimuli immediately applied to the muscles themselves.

The whole of this, as it may be truly called, reasoning in a circle, amounts to thus much; that in every animal there are certain parts subservient to functions absolutely necessary to the support of life, which act according to the necessities of the animal, uninfluenced by the will; and other parts which only act in conformity to the will; that, as far as we can judge, the brain appears so necessary to the communication of the will, that without its influence, the parts subject to the will, either cease to act, or act in an irregular manner; but that for the due action of parts subservient to the functions of life, a communication with the brain is not necessary.

All this, as we before remarked, is simple enough, if divested of the undefined terms, *organs* and *death*. Not that we object to either of these words as such; but we wish to know what a philosopher means, when he uses them. Mr. Hunter defines what he means by both, and carefully distinguishes between death and disorganization of certain parts; in consequence of which, though the actions necessary for the support of life can never be recovered, yet the individual parts for a time retain life. If the French philosopher and his English imitators had done so, they might have been intelligible.

Another difficulty they might also have avoided, had they distinguished between regular actions from customary stimuli, and mere contractions of muscles from unusual stimuli.

"Every one," says Dr. Wilson, "admits that the muscles of the limbs are muscles of voluntary motion, and that they are stimulated through the brain, and that the heart and blood vessels are muscles of involuntary motion, stimulated by the blood which is immediately applied to them." That the muscles of the limbs are subject to the will, and the muscles of the heart and blood vessels act without the will, cannot be questioned; but we are not so soon convinced that the latter are stimulated by the blood. "The flowing of the blood into the heart, (says Mr. Hunter, p. 148 and 149), has been assigned as a cause of its contraction; but even that will not account for it, although it will for many of its phenomena, yet not for all; for a local stimulus is too mechanical to produce all the variety attending the action of this viscus; it would not be attended with that regularity it has in health, nor that irregularity which we find in disease; neither could it ever stop, unless when *absolute universal death* * took place, nor resume its action if ever it did stop. We find, that those parts which have occasion for the immediate stimulus to produce action, have that action very irregular, as, for instance, the bladder of urine and the intestines," &c. It is unnecessary to transcribe any further. Our readers will perceive, this profound thinker and experimental philosopher, saw plainly that only one stimulus could be assigned for those actions which do not depend on the will, yet are necessary for life. That such a stimulus cannot depend on accidental circumstances, but must be constant, as the *necessity* which the constitution feels for the action of such parts. They can therefore only be referred to those laws which we may trace, but which it is in vain to attempt explaining. Those laws by which the growth and various changes of the animal are regulated, which are permanent, whilst only the customary actions

* This term Mr. Hunter has explained with much accuracy; have the other gentlemen explained what they mean by partial death?

tions of life are required, but which vary under every change, either healthy or diseased, in the condition of the animal. Those laws by which the absorption or augmentation of different parts successively take place, by which the late fœtus learns to breathe; the new formed fly to use its wings; by which adhesion takes place under inflammation, and suppuration sometimes follows previous to restoration of the diseased part.

Some of our readers may think we have dwelt too long on such a subject; but our wish was not only to given Dr. Wilson an opportunity of clearing himself of the charge of plagiarism, but to convince him, if possible, that there was nothing to steal. Others may accuse us in our blind partiality for Mr. Hunter, of giving Bichât too little credit. To these we can only answer, that we have taken him on the word of Dr. Wilson, and if we are mistaken, our sheets are open for such as wish to defend either.

Article 9.—*On the Cases in which the Use of Arsenic is indicated.* By James Jenkinson, Esq. Surgeon, Oxford.

In this very judicious paper, Mr. Jenkinson shows the folly of some writers, in expecting that a remedy is to cure every form of a disease which goes under the same name.

Article 10.—*Effects of Arsenic.* By G. N. Hill, Surgeon, Chester.

We hope that Arsenic, like many other valuable remedies, will not fall into disgrace by being too indiscriminately applied. These remarks, however, of Mr. Hill, may furnish some practical hints on the propriety of exhibiting other remedies at the time that arsenic is used.

Article 11.—*Account of the fasting Woman of Tetbury, who has at present lived above two Years without Food.*—By Benjamin Grainger, Member of the Royal College of Surgeons, London, and of the Royal Medical Society of Edinburgh.

We shall only take notice of such parts of this paper as we have not given at different times in our own Journal.

"An hypothesis has been started by some of my medical friends, that Ann Moore is an impostor. But what particulars of the case, according to this hypothesis, are impostures? Is the loss of appetite real or feigned? Was the tremendous pain after eating an imposture? The abolition of alvine excretion, and the suppression of urinary discharge, are they, according to this hypothesis, impostures? And yet I may with propriety remark, that there is such connection among the different particulars of the case, that the admission of one would go far to prove the existence of another."

"It is the opinion of some, that Ann Moore is nourished by materials which the body absorbs from the atmosphere. Air, surely, is not aliment; and water, which is contained in the atmosphere, is rather a vehicle of aliment than aliment itself. In health, when the functions of the system are in vigour, water will pass through through the organs of assimilation into the emunctories, without having undergone any important change. The oxygen which is consumed in the process of respiration cannot stand in the place of chyle, for the nourishment of the system. But it is questionable, whether a combination takes place between the oxygen of the atmospheric air, and the venous blood in the lungs, by respiration. Venous blood, it is said, differs only from arterial blood, in containing disengaged carbonaceous matter. The extreme branches of the pulmonary arteries expel this carbonaceous matter from the blood, and, in consequence, the venous blood of the whole body is transmitted from the pulmonary arteries into the pulmonary veins, *arterialized*."

We pretend not to determine what the nature of these changes in the lungs is; but this is certain, that they appear more necessary than food, as the animal suffers more from the privation of air than of food. Even a bad air appears to be more injurious than the worst kind of food; for with the greatest variety of aliment the body suffers without air, and with the advantage of pure air, a very meagre diet is sufficient to preserve health and strength. Nor are we quite satisfied that water is a mere vehicle of aliment, without being aliment itself; far less that it passes through the organs of assimilation without being materially altered. It is well known that fish will live without any other food than what they derive from distilled water, provided that water is changed often enough, and the surface exposed to the common air.

That the human race cannot live on air in common is very certain, for we believe no one ever tried the experiment very long without falling a sacrifice to it. But it is not easy to say what resources the constitution may have.

"With regard (says Mr. Granger) to the proximate cause of this disease, inedia prodigiosa,* I have nothing to offer. Thus far, I think, we may advance, that it is a disease of the whole system, and that the process of assimilation is suspended. The stomach being no longer an organ of digestion, food acts upon it as a foreign body, and creates that excessive pain referred to the region of the stomach, on the same principle as a particle of water, or of any other foreign body, would excite violent irritation in the windpipe. Whatever the cessation of the functions of the chylopoetic viscera may depend on, I do not think that dissection will throw any material light on the subject; for it is not easy to conceive, how such a morbid state of the viscera can satisfactorily be explained by a visible change of structure."

For our own parts we pretend not to determine what the proximate or remote cause of such a disease may be; but for those who wish to form theories, it seems rational enough to suppose the origin of

* The *anorexia mirabilis* of Sauvage has an extensive signification, not only including the disease at present under review, but also diseases in general, in which individuals have fasted long.

of such a state may be in the stomach, or some of the chylopoetic organs. If these are in such a state as to be unequal to their office, every other means of supporting life will be roused. Probably, dissection may throw no light upon the subject; for an organ may be diseased, without showing to our senses any marks of disease. On the whole, we forbear giving any remarks on so intricate a question, especially as we have no means of experiment, that is, of actually seeing, examining, and watching the subject. It ought, however, to be recollected, that the case is not without precedent, and that the correspondence of symptoms, between this and other events on record, entitle the whole to a grave consideration.

Article 12.—*Apology for the Cutting Gorget.* By W. Simmons, Surgeon, at Manchester.

This Apology is in Answer to Mr. Lawrence's Observations on Lithotomy. We are glad to take this opportunity to remark, that Mr. Simmons's paper appears to be candid and judicious. That it is liable to some objections is certain; but as our Journal contains an article in which these are remarked on with sufficient point, we shall content ourselves with transcribing a few passages which are particularly animadverted on.

"The ingenious Mr. L." says Mr. Simmons, "hath favoured the profession with a few "Observations on Lithotomy," in the last Number of your Journal, the scope of which is, to supersede the use of the *cutting-gorget* in that operation, and substitute, in its stead, the common scalpel, with a slight alteration. In order to strengthen his recommendation, he hath subjoined a case, which case, however, if not directly militating against his own doctrine, the advocates of the gorget may be inclined to think, is, at least, not greatly in favour of it. But, as the case is a solitary one, and the termination of it was fatal, I shall excuse myself from offering any further conjecture concerning it.

"In the operation of lithotomy, I have always employed the *cutting-gorget*, and with so much success, as to render me satisfied with it; though far from being inimical to improvement, where the change is really such, and founded upon just principles.

"In the introductory part of the observations before me, the author has taken occasion to remark on the uncommon success of Mr. Cheselden, as a lithotomist; and not wishing to deprive our countryman of any applause he may be justly entitled to, most willingly do I accord with this opinion. In justice to others, however, let it be observed, that dextrous as Mr. Cheselden undoubtedly was in performing this operation, he was no less fortunate to meet with so great a number of patients labouring of the stone, who were at the same time free from any disease of the kidnies and bladder, which might hurry on to a fatal termination; or whose habit did not predispose to inflammation, or tetanic affection, from which, even where the operation had been ably executed, and the patient was apparently out of danger from it, I have had occasion to witness a fatal event. "For

"For effecting the division of the prostate gland, and neck of the bladder, Mr. Cheselden employed the common scalpel; which, in process of time, yielded to the cutting-gorget, an instrument invented by Sir Cæsar Hawkins.

"To exalt either of these gentlemen at the expence of the other, is far from my intention; both were eminent in their day, and both are now no more, so that praise, or censure, is alike indifferent to them. But it is for the interest of science, that the question now agitated again, should be dispassionately canvassed; and, taking success for the criterion, I hope presently to shew, that whatever the merit of the scalpel in lithotomy may be, so far is it from being unfortunate that English surgeons did generally adopt the cutting-gorget, that it is still a valuable contrivance, and worthy of being retained in surgical practice. But to proceed:-

"The reason maintained by the author for his preference of the scalpel to the gorget, is scarcely consistent with his usual correctness. To suppose that Sir Cæsar Hawkins was ignorant of the anatomical structure of the parts concerned in the operation of lithotomy, would be entirely gratuitous. Nor is there more reason for suspecting the anatomical acquirements of many other surgeons, who have pursued his method of operating. Disclaiming, therefore, this mode of defence, I shall transcribe the passage on this subject for another purpose. "Sir C. Hawkins then (says the author, p. 138) may enjoy all the credit of introducing into one of the most difficult and dangerous operations of surgery, an instrument which can be employed by any individual, of any profession, or trade, who has the simple faculty of combining the motions of his two hands, so as to keep the gorget and staff together, as well as by the most skilful anatomist, and most experienced surgeon." With all proper deference to the author, this is, in my opinion, the highest commendation that ever was bestowed upon any surgical instrument; and how well it is merited by the gorget, my own experience of its use, for nearly twenty years, will bear irrefragable proof.—Yet, in stating my sentiments thus frankly, I hope not to be understood, as meaning to depreciate the importance of anatomical researches, or of dissecting-room surgery; nor am I insensible of the value of manual dexterity. But, to have rendered "one of the most difficult and dangerous operations of surgery," so simple, and easy of execution, as in the above quotation this author has described it to be, is so far from being a demerit, and on that account to be rejected, that it is a point of excellence, rarely, if ever attained, and ought to be aimed at in every other difficult and dangerous operation."

Mr. Simmons's paper concludes thus: "Such then is the *apology*, which I have to offer, for continuing to use the *cutting-gorget* in the operation of lithotomy. Yet, open to conviction, if by a still more ample experience, I should alter my opinion in favour of the common knife, I shall then without hesitation adopt it.

it. And I have ventured to graft my *apology* upon the observations of Mr Lawrence, because, though I may differ from him in opinion, on this occasion, yet, from the perusal of his other works, I have derived both pleasure and improvement."

The disease and operation, alluded to in this Controversy, are both of them deserving the highest consideration. For that reason we have admitted so many and such various remarks, hoping, that every advantage may be derived from the collision of opinions, arguments, and authorities.

Article 13.—*Case of very copious Hemorrhage from the Urethra, in Consequence of the Application of a Bougie, armed with the Argentum Nitratum, to a Stricture.* By Samuel Cooper.

This account is given with much candour, and concludes with the following paragraphs:

"The bleeding began about a quarter before eleven in the forenoon, and continued, more or less, till eight in evening.

"The case seems to me worthy of being recorded, on account of its exhibiting an example, in which the quantity of blood lost was unusually great, and in which the effects of the hæmorrhage on the constitution were such, as to forbid exposing the patient again to the same accident, lest it should bring on fatal consequences. The gentleman's health has been already so much reduced, that both Mr. Cline and Mr. Abernethy, who have been consulted, recommend no attempt to be made to remove the stricture, till the state of the constitution has been improved.

"Having always been an advocate for Mr. Home's plan of treating strictures, no one can suspect me of publishing this case with a view of bringing the method into disrepute. I still give a general preference to the employment of armed bougies, because they seem to me most efficacious; and the above case is the only one in which the fear of hæmorrhage has ever induced me to abandon this mode of treatment."

In the introduction Mr. S. Cooper remarks, "that it would be the height of imprudence ever to subject this particular patient again to another hæmorrhage of the same kind." We would wish the author to recollect, that, as it is impossible to ascertain *a priori*, what the consequence may be in any other subject, it may be adviseable in all to defer the use of the armed bougie, till every other attempt is found unsuccessful.

Article 14.—*Hints to young Practitioners, and Observations for the Benefit of those whom it may concern.*

As we trust few of our readers can be pleased with, or improved by these hints, and as our Journal is not intended for the use of such readers, we shall decline any further notice of this paper. That we may not be misunderstood, or suspected of misrepresentation, we shall transcribe the author's concluding quotation.

"——— *Abite pudor, verumque fidesque;*

*In vestrum subeantque locum fraudesque, dolique,*

*Insidiæque, et vis, et amor sceleratus habendi.*"

*A Prac-*

*A Practical Materia Medica, in which the various Articles are fully described, and divided into Classes and Orders, according to their Effects: their Virtues, Doses, and the Diseases in which they are proper to be exhibited, are fully pointed out. Interspersed with some Practical Remarks, and some select Formulæ: to which is added, a General Posological Table; intended principally for the Use of Students and Junior Practitioners.* 12mo. London, 1809.

It cannot be necessary to urge the convenience of a practical materia medica of the bulk of this before us. The executio. seems in all respects unexceptionable, as far as is consistent with the brevity of the whole.

" Medicines, says the author, in his introduction, are to the medical practitioner, what utensils or instruments are to the mechanic; unless he knows all their uses, he cannot succeed in his designs, and will be like a mechanic attempting to work without knowing the use of his instruments.

" If the time required to ascertain the qualities of one single medicine is considered, it must surprise and please us when we perceive the present improved state of the materia medica.

" The virtues of many medicines are very numerous; they agree with some constitutions only, from particular idiosyncrasy; they are specifics in some disorders, are proper at particular periods, and under peculiar circumstances. It is necessary to know the powers of each medicine, the symptoms requiring its exhibition, and the particular circumstances of constitution which may forbid it. If there was but one circumstance in diseases, the exhibition of remedies would be simple and easy; but there are almost always several diseased actions going on at the same time, nor is the disease similar in two persons; *v. g.* in common diarrhœa, although the indication of cure be the same, yet the same remedies are not often proper in two persons; in one there may be hard stools with mucus, in another, soft mucous stools, or colicky pains, or vomiting, or spasms, or dry skin, or skin natural.

" A medicine then is defined to be a substance or combination of substances which corrects the diseased action of a part, or of the whole, of the body. It may be taken from every one of the kingdoms, is modified much by art, and is often changed or much adulterated by druggists or dealers.

" The views in this volume are the following:

" 1. The *name* of the medicine and its synonims—and here it is much to be regretted that in most vegetable remedies, the generic name is at present only used.

" 2. *The more evident properties:* i. e. the physical, and to a certain degree the chemical. By the *physical* properties are meant, those which it has in common with other substances, as figure, extent. All drugs have in a great measure the same chemical properties.

" 3. The *effect* produced on the human frame, both in health and disease.

" 4. The

"4. *The manner in which it is supposed to act.* Medicines have distinct principles; their powers operate more on one part of the system than another, as the stomach, skin; and every medicine produces two kinds of events, that is, their primary and secondary operation; *v. g.* opium is at first a stimulant, and afterwards a narcotic.

"5. Under what restrictions it may be given.

"6. Its various forms, and the compounds into which it enters. The multiplicity of the articles, and their great difference point out the necessity of some *arrangement;* but as to the manner, this has varied. The general principle on which these arrangements are formed, is founded on the evident or external character, or on the medicinal effect."

After enumerating the various divisions adopted by different writers, the author prefers the last, or that founded on medicinal effects. This is certainly the most convenient for the physician, who has little else to do with any article, but to learn its effect on the human body; but for the apothecary, we conceive any other arrangement might be better. However, as the greater part of that branch of the profession have now a medical education, probably the present form may be the most extensively useful.

---

*A plain Statement intended for the Information of the Public, on the Comparative Advantage of the Cow-pox and Small-pox Inoculations, with an Abstract of the Report of the Royal Colleges of Physicians and Surgeons, on the Cow-pox.* By T. Smith, M. D. late Physician to the Nottingham General Hospital, and Fellow of the Royal Medical Society of Edinburgh, 8vo. London 1809.

Nothing very new can be expected from a publication professing all that is contained in the title page. If the object is merely to afford a cheap summary of the arguments in favour of vaccination, we apprehend that this has been already done in a more pointed and compact form by the "Reports of the Colleges, with remarks," and sold for the benefit of the small-pox hospital.

---

*A Treatise explaining the Impossibility of the Disease termed Hydrophobia being caused by the Bite of any Rabid Animal.* By William Maryan, Surgeon, Rotherhithe Wall, 8vo, London, 1809.

This is an ingenious little pamphlet, but to us by no means satisfactory. Without disputing or even doubting the accuracy of every case as stated by our author, we still remain convinced from what has occurred to our own observation, that hydrophobia may arise from the bite of a rabid animal.

"A disease, says Mr. Maryan, which may be considered coeval with hydrophobia, *witchcraft*, did once exist over the whole of that now enlightened part of the world called Europe; but since the introduction of printing, the progress of the Arts, and the increase of learning in this quarter of the globe, that disease has has been abolished; and, I have no hesitation in declaring it to be my firm belief, that when the nature of the disease, to which I am now calling the attention of the world, becomes more generally understood, and more fully investigated, it will be found to possess as little foundation as that above alluded to."

In answer to this, we cannot help remarking, that the suspicion of witchcraft, or which is the same thing, of fascination, is much older than of hydrophobia from a rabid animal; and also that whilst the suspicion of the first has gradually gone into disuse with the improvement of human knowledge, the existence of the latter is become daily more confirmed.

We are far from wishing to undervalue the author's good intention, industry, or faithfulness. We particularly regret that we are obliged to admit with him, that no true diagnostic symptom of hydrophobia has hitherto been discovered in dogs, or any other animal, (excepting man.) The difficulty of swallowing certainly does not occur in the canine species, nor has it been remarked in cats.

The following case being authenticated by the names of the author and patient, is very well worth recording with as much publicity as possible.

"The Case of Mr. Castleman, of the Grove House, Camberwell.—Mr. Castleman was bitten by a mad dog in the spring of the year 1771, as he was attending his dogs to take the air in coupling time—A large dog sprang upon one of his, from a hedge, with great fury: he ran to beat him off, when this assailant seized him fast by the leg, and severely lacerated it; being, however, at last disengaged, he returned home, and his mind became considerably alarmed for the consequences. The dog, which belonged to a farmer in the neighbourhood, was observed by his master, on his reaching home, who, fearful lest he might be going mad, had given orders for him to be secured by a strong chain; this was effected by fastening him to a tree in the orchard. Every thing within his reach became the subject of attack, and at the expiration of two days he died. Three weeks had elapsed, when Mr. Castleman, finding himself getting worse, sent for a Physician, who resided in the neighbourhood, and who, on his arrival, enquired if Mr. C. had been bitten by a mad dog; on Mr. Castleman replying in the affirmative, the physician concluded immediately that that was the cause and origin of his illness; mercurials were therefore, both internally and externally, administered, to destroy the supposed *Virus*; Mr. C's spirits every day grew worse, nor could he obtain rest, even at night; no—

"Tir'd Nature's sweet restorer,
"Balmy sleep———"

was a stranger to his eyelids; his appetite he lost, and irritability and debility had taken place of tranquillity and health.

"In this situation the physician of the adjoining village was consulted, in addition to the former one; they were both of one opinion, viz. that the disease was occasioned by the bite of a mad dog.

—Mr.

—Mr. Castleman now concluded that death must ensue; but, hearing of Dr. Hamilton's "Treatise on Madness," he caused it to be purchased; he there learned, that people had been affected, and that death had been the consequence, even though a space of ten or fifteen years had elapsed since the cause. The perusal and study of Dr. Hamilton's treatise very much increased his distress, and he gave himself up for lost. In this state he continued for three years, continually and deeply impressed with the idea, that he should bite some one or other of his friends, and entail on them the dreadful calamities under which he was then suffering; the certainty that this would be the case, was ever uppermost in his thoughts, producing in him the greatest despair that it is possible for human nature to picture or describe. It was in this stage of his disorder that a physician from London was consulted, in addition to, and in the presence of, those before mentioned; he confirmed their opinion, that their patient was labouring under hydrophobia. To ascertain this, a glass of water was brought, at the sight of which, Mr. Castleman declares, he was as much alarmed as if a highwayman had stood before him with a drawn sword, and was going to run him through the body; from all appearances therefore, they concluded, not only that hydrophobia had taken place, but that it was very far advanced. They retired to an adjoining room, to consult what was to be done. Mr. C. anxious to know their real sentiments, listened to their conversation, and indeed, with the greatest attention and interest. He heard one of them observe, that he thought the medicine too powerful; and another, that in desperate cases, like the present, desperate remedies only would be likely to succeed. Mr. C. himself, had but a confused idea of the word hydrophobia, conceiving, that whenever it did take place, it would act something like a two-edged sword, and immediately deprive him of existence. When the physicians returned into the room, he enquired how the hydrophobia would act when it attacked the patient. They informed him it would produce a dread, or fear of water, and added, which you seem to have on you, and if you could swallow a little water, it would be much in your favour.

"The physicians left him, and when they were gone he reflected on their observations, and began to conceive he had no dislike to water; he sent for some, and found that he had no difficulty in swallowing it. When his medical attendants called the next day, he related the circumstance to them; they observed, it was a very favourable symptom, and if he could take liquids frequently, there would be no danger. Mr. C. did take liquids frequently, and very speedily recovered; and he has since often declared that his recovery was owing to his being made completely to understand the meaning of the word hydrophobia.

"The painful sufferings of Mr. Castleman, which he endured uninterruptedly for three or four years, and his great partiality for dogs and sporting, have induced him to pay particular attention to the disease of the canine species, and, in no stage of madness in dogs, could he perceive they ever refused water, or had any difficulty in swallowing: he declares it, therefore, to be his firm opinion that dogs cannot convey any disease to the human race; he is further strengthened in this opinion by his having been since bitten, twenty times at least, by dogs in the same disease, without its ever producing either mental or bodily complaints.

"Mr. Castleman has frequently attended people in the neighbourhood, where there have been mad dogs; and when any person has been bitten, he has always prevented any disease taking place by convincing the sufferers of the perfect safety of their situation; telling them that there was not any danger; acquainting them of the utter impossibility of dogs communicating disease to man; and by endeavouring, in every possible way, to dispossess the mind of fear."

Satisfied as we are that unfounded apprehension has embittered many a valuable life, yet we cannot admit this as a proof that there are no such diseases.

---

*Analysis of the Carbonated Chalybeate, lately discovered near Stow; with Observations on the Effects of Carbonic Acid and Nitrogen Gas on the Animal Economy, &c. with Extracts from some of the best Authorities we have, relative to the Use of Chalybeates; to which is subjoined a Glossary of the Technical Words necessarily made Use of in the Work.* By R. FARMER, 8vo, London, 1808.

WE announce this more as medical news, than from any knowledge of our own. The chalybeate water of Stow, if this analysis is correct, contains considerably more iron and carbonic acid gas than either Tunbridge, Cheltenham, or even Spa.

We conceive the glossary announced in the title page must be omitted, at least in our copy.

